# A quarter of young Japanese women are underweight: thin-ideal internalization and self-esteem mediate exercise habits and body satisfaction, but different mechanisms with normal-range weight

**DOI:** 10.1186/s12889-025-24537-8

**Published:** 2025-11-20

**Authors:** Yuka Murofushi, Shinji Yamaguchi, Yasuyo Yoshizawa, Yoshifumi Tamura

**Affiliations:** 1 Faculty of Health and Sports Science, Juntendo University, Hiraka-gakuendai, Inzai-shi, Chiba 270-1695 Japan; 2 Graduate School of Health and Sports Science, Juntendo University, Hiraka- gakuendai, Inzai-shi, Chiba Japan; 3 Division of Public Health, Department of Hygiene and Public Health, School of Medicine, Tokyo Women’s Medical University, Shinjuku-ku, Tokyo, Japan; 4 Faculty of International Liberal Arts, Juntendo University, Bunkyo-ku, Tokyo, Japan; 5 Department of Metabolism and Endocrinology, Juntendo University Graduate School of Medicine, Bunkyo-ku, Tokyo, Japan; 6 Department of Sports Medicine and Sportology, Juntendo University Graduate School of Medicine, Bunkyo-ku, Tokyo, Japan

**Keywords:** Exercise habits, Body satisfaction, Sociocultural attitudes toward appearance, Self-esteem, Underweight, Normal-range weight

## Abstract

**Background:**

In Japan, approximately 20–25% of young women are underweight, highlighting a significant social issue with considerable future health risks. This generation is strongly influenced by the internalization of thin ideals, leading to a belief that being thin equates to beauty and a misconception that it equates to health. Recent studies have revealed that young women with insufficient physical activity and low dietary intake exhibit metabolic profiles similar to those of obese individuals, putting them at increased risk for developing diabetes in the future. Addressing these issues requires a multifaceted approach, with stable exercise habits crucial for enhancing body satisfaction by reducing the internalization of thin ideals and improving self-esteem. Given this context, it is essential to investigate the effects of exercise habits, particularly in underweight women. Therefore, this study investigates how exercise habits influence body satisfaction by mediating thin-ideal internalization and self-esteem, comparing these effects between underweight and normal-range weight women.

**Methods:**

The study surveyed 400 young women aged 18–29 who are underweight and 189 with normal-range weight across Japan, assessing their exercise habits based on the Ministry of Health, Labour and Welfare’s National Health and Nutrition Survey criteria (exercising at least twice a week for over a year, with sessions lasting ≥ 30 min), subjective body satisfaction, sociocultural attitudes toward appearance, and self-esteem. Data analysis was conducted using the PROCESS macro for SPSS, which allowed for mediation analyses to test indirect effects. Thin-ideal internalization and self-esteem were analyzed as mediators of the relationship between exercise habits and body satisfaction. Mediation analyses were performed separately for underweight and normal-range weight groups to identify differences in the mechanisms underlying these relationships.

**Results:**

Approximately 50% of participants in both groups reported regular exercise (average 2.5 days/week). In the underweight group, exercise habits fully mediated the relationship between reduced thin-ideal internalization, increased self-esteem, and improved body satisfaction. In the normal-range weight group, exercise habits reduced thin-ideal internalization and improved body satisfaction but had no significant effect on self-esteem.

**Conclusions:**

The results highlight that the pathways through which exercise habits affect body satisfaction differ according to body mass index. For underweight women, reducing thin-ideal internalization and enhancing self-esteem through exercise habits is crucial. In contrast, interventions directly boosting self-esteem may be more effective for women with normal-range weight. These findings clarify the distinct roles of exercise habits in influencing body satisfaction for underweight and normal-range weight women in Japan.

## Introduction

In Japan, approximately one in four to five young women are underweight, a serious social issue that has steadily increased since the 1990 s [1, 2]. Initially around 12–13%, this trend now affects 20–25% of young women aged 10–20 [1, 2], far exceeding the prevalence of underweight in impoverished South Asian regions [3]. Cultural shifts during the 1990 s, driven by the idol industry, popularized thinness as a societal norm by publishing idols’ heights and weights in teen magazines [4]. More recently, the trend of ‘Cinderella weight,’ largely fueled by social media, has encouraged young women to aim for a body weight approximately 10 kg below the recommended BMI [5].

Distorted body image and dissatisfaction, which can lead to extreme thinness among young women, may result from the internalization of thin ideals and low self-esteem [6, 7]. Media-driven messages about beauty foster body dissatisfaction [6], lower self-esteem, and exacerbate dissatisfaction with body image [8]. Lookism, which values individuals based on physical appearance [9], might reinforce the belief that ‘thin is beautiful’ and drive thin women to further weight loss.

According to the BMI standards, both underweight (BMI < 18.5 kg/m²) and severe obesity (BMI > 35 kg/m²) significantly increase health risks, prompting various etiological and preventive strategies [10]. These include strategies for promoting exercise habit to improve body image and body satisfaction [11–13]. However, most of these studies focus on normal-range weight (BMI 18.5–25 kg/m²) or obesity (BMI > 25 kg/m²) populations, leaving a gap in research on underweight individuals. Underweight women tend to evaluate their body image based on weight and shape [14], suggesting that the mechanisms underlying body satisfaction in this group may differ from other BMI groups. Japanese women, in particular, generally have low self-esteem and high social anxiety, which are associated with low physical self-esteem [7].

In light of these findings, understanding the impact of sociocultural attitudes toward appearance (thin-ideal internalization) [15] and self-esteem [16] on body satisfaction through exercise habits is crucial for developing appropriate health promotion strategies for this population.

### Health concerns of underweight women

Globally, severe obesity affects approximately 6% of men and 9% of women [3]. In high-income countries like the United States, over 60% of adults are overweight or obese, while in Japan, this figure is only 27% [17]. Severe obesity (BMI > 35 kg/m²) poses health risks, including its association with type 2 diabetes, cancer, and cardiovascular disease [18]. While obesity poses significant health risks globally, underweight individuals, particularly young women, face distinct but equally critical health challenges.

The lack of body type inclusivity diminishes the quality of life for women and increases health problems related to being underweight, especially among young women. These include menstrual irregularities, infertility, anemia, anorexia nervosa, chronic fatigue, lowered self-esteem, psychological stress, and increased mortality risk [19]. Moreover, thinness at a young age increases the risk of osteoporosis, bone fractures, sarcopenia, and lifestyle-related diseases in the future. Recent studies show that young women who eat little and engage in insufficient physical activity face a risk of diabetes comparable to that of obese individuals [20, 21]. These challenges are critical health issues with implications for women’s empowerment, declining birth rates, and the health of future generations [19–21]. In particular, promoting well-balanced exercise habits tailored to individual needs is critical for improving both physical and psychological well-being among underweight young women.

### The role of exercise habits

Exercise habits positively influence body satisfaction, with psychological variables such as self-esteem and self-efficacy contributing to this effect. Regular exercise has been shown to enhance life satisfaction and happiness while alleviating depression [22, 23]. Consequently, it plays a crucial role in maintaining psychological health and well-being, while also mitigating health risks. Previous research has demonstrated that structured exercise interventions—planned and systematically implemented programs—effectively enhance body satisfaction and improve subjective evaluations related to body image [24]. Additionally, studies have reported that resistance training can significantly improve various aspects of body image, including body satisfaction, appearance evaluation, and social physique anxiety [25]. However, excessive exercise can lead to decreased body satisfaction and self-esteem in young women [26]. Therefore, it is important to maintain moderate rather than excessive exercise habits. Particularly among young underweight women, it is necessary to examine whether maintaining appropriate exercise habits tailored to individual needs can improve thin-ideal internalization [15], thereby enhancing self-esteem and improving body satisfaction.

### Thin-ideal internalization, self-esteem, body satisfaction

Cognitive and emotional evaluations that undervalue body image and body satisfaction might hinder the accurate assessment of self-esteem. Self-esteem refers to how a person feels about themselves and includes evaluations about their abilities and worth [16]. High self-esteem does not mean feeling “very good” about oneself but involves a sense of self-acceptance as “good enough” [27]. Conversely, low self-esteem reflects self-denial, dissatisfaction with oneself, self-contempt, and lack of self-respect [27]. Approximately 80% of Japanese teenagers perceive themselves as overweight despite not being so and prefer a thinner body type over a healthy one, leading to a strong desire to lose weight [28]. The gap between body image ideals and reality may affect self-esteem. The extensive internalization of sociocultural body image standards significantly predicts body dissatisfaction [29], and the gap between the ideal and reality may affect self-esteem, particularly among Japanese women.

For instance, media-promoted beauty ideals can lead to body dissatisfaction and lower self-esteem, often associated with dissatisfaction with body image [8]. Social comparison based on appearance is common on social media, which increases the risk of eating disorders [30]. Photo-related activities focusing on appearance can decrease body satisfaction [31]. Particularly among young women, body image disturbance strongly correlates with the onset of eating disorders [31]. Misinterpreting societal norms regarding attractive body image can lead to problematic behaviors, highlighting the effectiveness of intervention strategies to address these issues [32]. Furthermore, low body satisfaction can lead to unhealthy eating attitudes, dieting behaviors, weight control efforts, and reduced physical activity levels [33]. Thus, low body satisfaction might fail to motivate healthy weight management behaviors. While avoiding the internalization of the thin ideal from the media and increasing body satisfaction is important, these effects may vary by BMI category, even among young women.

### Characteristics of underweight young women

Among young Japanese women, body satisfaction differs significantly between underweight and normal-weight individuals, with 19.5% of underweight women reporting dissatisfaction compared to 42.9% of normal-weight women [20]. Further analysis indicates that underweight women had lower average birth weights. While both groups have an average BMI and ideal BMI of around 17.5 kg/m², only the underweight group matches their ideal BMI. Additionally, 10% of underweight women are concerned about gaining weight compared to 45% of normal-weight women, suggesting that normal-weight women may be more dissatisfied with body changes. Although self-esteem does not differ, sociocultural pressure related to appearance is significantly higher among normal-weight women. This indicates that underweight women may experience less discrepancy between their ideal and actual body shape, contributing to higher body satisfaction [34]. These findings suggest that thin-ideal internalization and self-esteem may differ across BMI categories. For example, the influence of regular exercise on body satisfaction might have significant mediation effects, particularly in underweight women. Nevertheless, further exploration of these relationships is needed.

### Present study

**Fig. 1:**
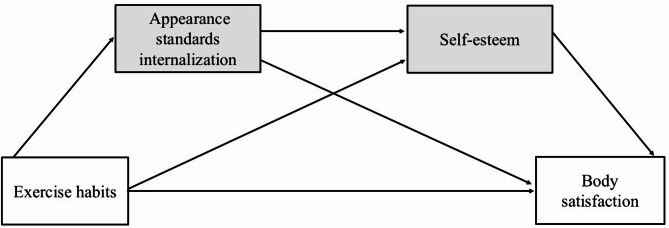
Hypothesized mediation model: The influence of exercise habits on body satisfaction mediated by sociocultural attitudes toward appearance and self-esteem

This study aims to examine how exercise habits influence body satisfaction through the mediation of thin-ideal internalization and self-esteem among young, underweight Japanese women (Fig. 1). Additionally, it aims to determine whether these mechanisms differ from those observed in women with normal-range weight. In this context, the study will examine the differences in exercise habits, body satisfaction, thin-ideal internalization, and self-esteem between the two groups, and analyze the relationships between these variables across BMI categories. This approach will provide insights into supporting the development of healthy body image across different BMI groups and offer guidance on how exercise habits contribute to mental and physical health.

## Materials and methods

### Study design

This study employed a cross-sectional design to explore the relationships between exercise habits, body satisfaction, sociocultural attitudes, and self-esteem among underweight and normal-range weight young women in Japan. The design facilitated group comparisons (underweight vs. normal-range weight) and mediation analyses to investigate the potential underlying mechanisms.

### Participants

This study recruited underweight and normal-range weight women aged 18–29 across Japan through an online research company in Japan. Exclusion criteria included current hospitalization for any illness and participation in sports requiring weight loss. The goal was to obtain approximately 250 valid responses from each group, anticipating some missing data. After applying exclusion criteria and obtaining informed consent, the final valid responses included 400 underweight women (mean age: 25.0 ± 3.0 years; mean BMI: 17.43 ± 0.92 kg/m²) and 189 normal-range weight women (mean age: 25.5 ± 3.0 years; mean BMI: 20.65 ± 1.55 kg/m²). Given that 20–25% of young women in Japan are underweight [20], a larger dataset was collected for this group to ensure sufficient representation and statistical power. This approach ensured that the study results accurately reflect the population characteristics and provide reliable insights into the underweight group. The study adopted guidelines for testing mediation effects [35] to ensure adequate statistical power. The sample size significantly exceeded the minimum required to achieve a power of 0.8, allowing the identification of hypothesized mediation effects.

### Survey methods, period, and ethical considerations

This study obtained approval from the Research Ethics Committee of the first author’s institution (IRB No.: 2025–66). The survey was conducted in May 2023 through an online research company in Japan. Registered panel members were invited to participate. The average time required to complete the survey was approximately 15 min. The survey page explained the research purpose and procedures, emphasizing voluntary participation and privacy protection. It was stated that participants’ data would remain anonymous and that they could withdraw from the study without any disadvantage. Informed consent was presented to all participants at the beginning of the survey, and only those who agreed proceeded to answer the main survey.

### Measurements

#### Participant characteristics and exercise habits

The survey for this study was based on the questionnaire used by Murofushi et al. [20]. Participants were asked to provide information on age, current height and weight, and BMI (weight in kilograms divided by the square of height in meters). Exercise habits were assessed using the National Health and Nutrition Survey [36] criteria to facilitate comparisons with existing data from Japan. *Exercise habits* were defined as “engaging in exercise for at least 30 minutes per session, at least twice a week, and maintaining this routine for at least one year.” Participants were asked to indicate whether they met this definition by selecting either ‘Yes’ or ‘No.’ Those who answered ‘Yes’ regarding their exercise habits were further asked about the average weekly exercise days and the duration per session (in minutes). According to the results of the “2023 Public Opinion Survey on Sports Participation” conducted by the Japan Sports Agency, the percentage of young women with regular exercise habits is 17.7% for those in their teens and 13.6% for those in their twenties [37]. Respondents were also asked to respond to multiple ‘Yes’ or ‘No’ questions regarding their reasons for exercising, including: “health and fitness”; “feeling inadequate in physical exercise”; “fun or distractions”; “beauty or obesity reduction”; “stress relief”; “spiritual cultivation or training”; “improving my record or ability”; “socializing with friends and colleagues”; “spending time with family”; and “club activities.”

#### Subjective body satisfaction question

In Japan, valid methods exist to measure body satisfaction and body image, such as the Japanese version of the Body Shape Questionnaire [38] and the Stunkard’s Figure Rating Scale [39], which do not require translation. However, these tools primarily assess body recognition and image rather than comprehensively measuring body satisfaction, which is the central focus of this study. Recognizing the lack of a standardized scale specifically for body satisfaction, such as the one developed by Slade et al. [40], we created a single-item measure tailored to capture participants’ overall perception of their body shape comprehensively. This measure was developed based on the question proposed by Murofushi et al. (2023) [20] to minimize respondent burden while effectively assessing body satisfaction. A focus group comprising experts from diverse fields—sports medicine, sports psychology, athletic training, kinesiology, healthy life expectancy, and health psychology—collaboratively designed the question. To ensure face and content validity, the questionnaire underwent rigorous evaluation and review. Participants rated their satisfaction with their body shape using a 6-point Likert scale (Table 1), where higher scores indicated greater satisfaction. In Murofushi et al.’s study (2023) [20] (*N* = 5,901), body satisfaction was categorized as Dissatisfied, Normal, or Satisfied, yielding the following results: underweight (21.4%/68.7%/9.9%), normal-range weight (44.6%/52.3%/3.0%), and obese (85.0%/14.6%/0.4%).

**Table 1:** Subjective body satisfaction question

Body Satisfaction Question	
How satisfied are you with your body and body shape? ‘Body shape’ includes all aspects of your physical appearance—*weight*, *body fat percentage*, *muscle mass*, and *proportions*. Select the option that best describes how you feel currently.	
1	Very dissatisfied: Constantly concerned about your body shape, affecting your daily life.
2	Dissatisfied: Frequently concerned about your body shape, making it difficult to feel confident
3	Somewhat dissatisfied: Occasionally concerned about your body shape but it does not significantly impact your daily life.
4	Somewhat satisfied: Sometimes concerned about your body shape but generally satisfied.
5	Satisfied: Not particularly concerned about your body shape, feeling confident in daily life.
6	Very satisfied: Extremely satisfied with your body shape, having no concerns.

Prior research has well established the validity of single-item measures, particularly when assessing specific and well-defined concepts [41]. Single-item measures are considered appropriate when the measured content is straightforward and clear. Therefore, this study aimed to obtain simple and reliable data while balancing precision and respondent burden.

#### Sociocultural attitudes towards appearance questionnaire-3 JS

This scale measures the internalization and awareness of the thin-ideal. The original Sociocultural Attitudes Towards Appearance Questionnaire-3 (SATAQ-3), developed by Thompson et al. (2004) [15], assesses how individuals internalize societal appearance standards and feel pressured to conform. It consisted of 30 items across four subscales: information (availability and importance of societal appearance standards), pressures (pressure to conform), internalization-general (adoption of societal standards as personal standards), and internalization-athlete (internalization of athlete-specific standards). Items are scored on a 5-point Likert scale, from completely disagree [1] to completely agree [5]. This study used the brief 12-item Japanese version, SATAQ-3 JS [42], with total scores ranging from 12 to 60. Higher scores indicate a stronger tendency to internalize societal appearance standards and pressures. The study’s evaluation was based on the total score rather than subscale scores.

#### Rosenberg self-esteem scale

The Rosenberg Self-Esteem Scale (RSE) [16] has been widely used to measure overall self-esteem. It includes 10 items forming a single factor, each representing a positive or negative self-assessment. Items include statements like “I feel that I’m a person of worth” and “I take a positive attitude toward myself,” rated on a Likert-type scale of agreement. This study used the Japanese version (RSE-J) [27]. While the original uses a 4-point Likert scale, the validated Japanese version employs a 5-point scale, which this study adopted. Responses range from strongly disagree [1] to strongly agree [5], including a mid-point [3]. Negative items are reverse-scored, and the total score reflects overall self-esteem. The score ranges from 10 to 50, with higher scores indicating higher self-esteem levels.

### Statistical analysis

All analyses were conducted using SPSS 29 software (IBM, Tokyo, Japan). First, the reliability of thin-ideal internalization (SATAQ-3 JS) and RSES-J was assessed by separately calculating Cronbach’s alpha coefficients for the underweight and normal-range weight groups. Internal consistency was evaluated using the following criteria: α ≥ 0.9: Excellent, 0.8 ≤ α < 0.9: Good, 0.7 ≤ α < 0.8: Acceptable, 0.6 ≤ α < 0.7: Questionable, α < 0.6: Poor [43].

To examine the study’s first objective, the measured variables between the underweight and normal-range weight groups were compared using independent samples t-tests, and the effect size (*d*) was calculated. The interpretation of effect size (*d*) was as follows: *d *≤ 0.2, small effect; *d* ≈ 0.5, medium effect; and *d ≥* 0.8, large effect (Cohen, 1988). For comparing the extent of exercise habits between groups, cross-tabulation and χ^2^ tests were conducted, and Cramer’s V statistic was reported as the effect size. The interpretation of V coefficients was as follows: 0.05, weak; 0.10, moderate; 0.15, strong; 0.25, very strong [44].

For the second objective, the relationships between variables within the underweight and normal-range weight groups were examined. Pearson’s correlation coefficients were calculated among exercise habits (presence or absence), thin-ideal internalization, self-esteem scale, and body satisfaction scores to evaluate the strength of the correlations. The interpretation of correlation coefficients was as follows: 0.10 to 0.29 as weak correlation, 0.30 to 0.49 as moderate correlation, and 0.50 or above as strong correlation [45, 46].

For the third objective, the mediating effects of thin-ideal internalization (M1) and self-esteem (M2) on the relationship between exercise habits (X) and body shape satisfaction (Y) were examined. The analysis was conducted using PROCESS [47] v4.2 SPSS Macro, setting Model 6, and by calculating the Bootstrapped Standard Error (BootSE) employing the Bootstrap method (10,000 samples). All effects were estimated with 95% confidence intervals. All direct path coefficients were reported as partially standardized estimates (unstandardized B with SE, CI, and p values). In addition, the completely standardized indirect effect (CSIE) was interpreted according to the criteria of 0.01 for small, 0.09 for medium, and 0.25 for large effects [48].

## Results

### Scale reliability, descriptive statistics, and correlation analysis

First, a reliability analysis of the scales for each group, underweight and normal-range weight, was conducted. The underweight group’s thin-ideal internalization showed excellent reliability (α = 0.93), and the Self-Esteem Scale showed good reliability (α = 0.86). The normal-range weight group’s thin-ideal internalization also showed excellent reliability (α = 0.94), and the Self-Esteem Scale was good (α = 0.86).

Next, exercise habits were reported by 51.7% (*n* = 207) of the underweight group and 50.3% (*n* = 95) of the normal-range weight group, with no significant differences between groups, while the effect size was weak (χ^2^ = 0.113 [1], *p* =.736, V = 0.014). A comparison of reasons for having an exercise habit showed no difference between the underweight and normal-range weight groups in any of the categories (Table 2). For both groups, the most common reasons for exercise habits were “health and fitness” and “perceived lack of exercise” (80% range), as well as “beauty enhancement and obesity reduction” (60% range).

**Table 2:** Chi-square test comparison results for reasons related to exercise habits (multiple choice).

Items	Underweight (*n* = 210)	Normal-range weight (*n* = 92)	χ^2^	df	*p*	V
Health and fitness.	87.1%	82.1%	1.517	1	0.218	0.071
Perceived lack of exercise.	81.6%	82.1%	0.009	1	0.923	0.006
For fun, distractions.	72.0%	66.3%	0.999	1	0.318	0.058
Beauty enhancement and obesity reduction.	61.4%	68.4%	1.405	1	0.236	0.068
To relieve stress.	52.7%	48.4%	0.468	1	0.494	0.039
For spiritual cultivation or training.	31.4%	28.4%	0.273	1	0.601	0.030
To improve my record or ability.	27.5%	30.5%	0.286	1	0.593	0.031
Socializing with friends and colleagues.	25.1%	26.3%	0.049	1	0.825	0.013
Spending time with family.	16.9%	24.2%	2.238	1	0.135	0.086
Club activities.	9.7%	12.6%	0.606	1	0.436	0.045

*Note*. Data are restricted to participants who reported having exercise habits (exercise at least twice a week for at least 30 min each time for at least one year)

Table 3 shows the means, standard deviations, and group comparisons (*t*-tests) of the measured variables in the underweight and normal-range weight groups. Significant differences were observed in body satisfaction, with the underweight group (*M* = 3.36, *SD* = 1.09) scoring higher than the normal-range weight group (*M* = 2.67, *SD* = 1.07), indicating a medium effect (*d* = 0.64). Furthermore, significant differences were observed in exercise duration per session (minutes), with the underweight group (*M* = 80.20, *SD* = 71.25) exercising longer than the normal-range weight group (*M* = 65.55, *SD* = 42.44), indicating a small effect (*d* = 0.23).

**Table 3:** Comparison of questionnaires among underweight and normal-range weight women (t-test)

Items	Underweight (*n* = 400)		Normal-range weight (*n* = 189)		t	df	*p*	95% CI	d *
	M	SD	M	SD					
Exercise habits per week (days) ^a^	2.83	1.97	2.42	1.54	1.96	228.80	0.051	− 0.01-0.82	0.22
Exercise duration per session (minutes) ^a^	80.20	71.25	65.55	42.44	2.22	280.38	0.027	1.68-27.63	0.23
Body satisfaction ^b^	3.36	1.09	2.67	1.07	7.23	587.00	< 0.001	0.50-0.88	0.64
Sociocultural attitudes towards appearance ^c^	33.05	13.03	34.44	14.14	−1.18	587.00	0.239	−3.72-0.93	0.10
Self-esteem ^d^	28.64	7.85	29.20	7.63	−0.82	587.00	0.414	−1.91-0.79	0.07

Note. *M* Mean, *SD* Standard Deviation

*An effect size (*d*) ≤ 0.2, small effect; 0.5, medium effect; and 0.8, large effect (Cohen, 1988)

^a^50.3% (*n* = 207) of underweight and 51.7% (*n* = 95) of normal-range weight respondents who have an exercise habit. Possible range scores for

^b^1–6

^c^12–60

^d^10–50

The correlation analysis found a significant, moderately positive correlation between body satisfaction and self-esteem (*r* =.311, *p* <.001) in the underweight group **(**Table 4a). A significant weak negative correlation was also observed between thin-ideal internalization and self-esteem (*r* = −.233, *p* <.001). In the normal-range weight group, a significant, moderately positive correlation was found between body satisfaction and self-esteem (*r* =.287, *p* <.001) (Table 4b). No significant correlation was observed between thin-ideal internalization and self-esteem.

**Table 4:** Descriptive statistics and zero-order correlations

Items	M	SD	1	2	3	4
Underweight group						
1. Body satisfaction ^a^	3.36	1.09	1			
2. Exercise habits ^b^	0.52	0.50	0.014	1		
3. Sociocultural attitudes towards appearance ^c^	33.05	13.03	− 0.146**	− 0.154**	1	
4. Self-esteem ^d^	28.64	7.85	0.311***	− 0.108*	− 0.233***	1
*Normal-range weight group*						
1. Body satisfaction ^a^	2.67	1.07	1			
2. Exercise habits ^b^	0.50	0.50	-.031	1		
3. Sociocultural attitudes towards appearance ^c^	34.44	14.14	-.244***	-.162*	1	
4. Self-esteem ^d^	29.20	7.63	.287***	.004	-.030	1

*Note*. *M* Mean, *SD* Standard Deviation

* *p* <.05

** *p* <.01

*** *p* <.001

Possible range scores for ^a^1–6; ^b^0 or 1 (0: no/1: yes); ^c^12–60; ^d^10–50

### Mediation analysis

#### Underweight group

**Fig. 2:**
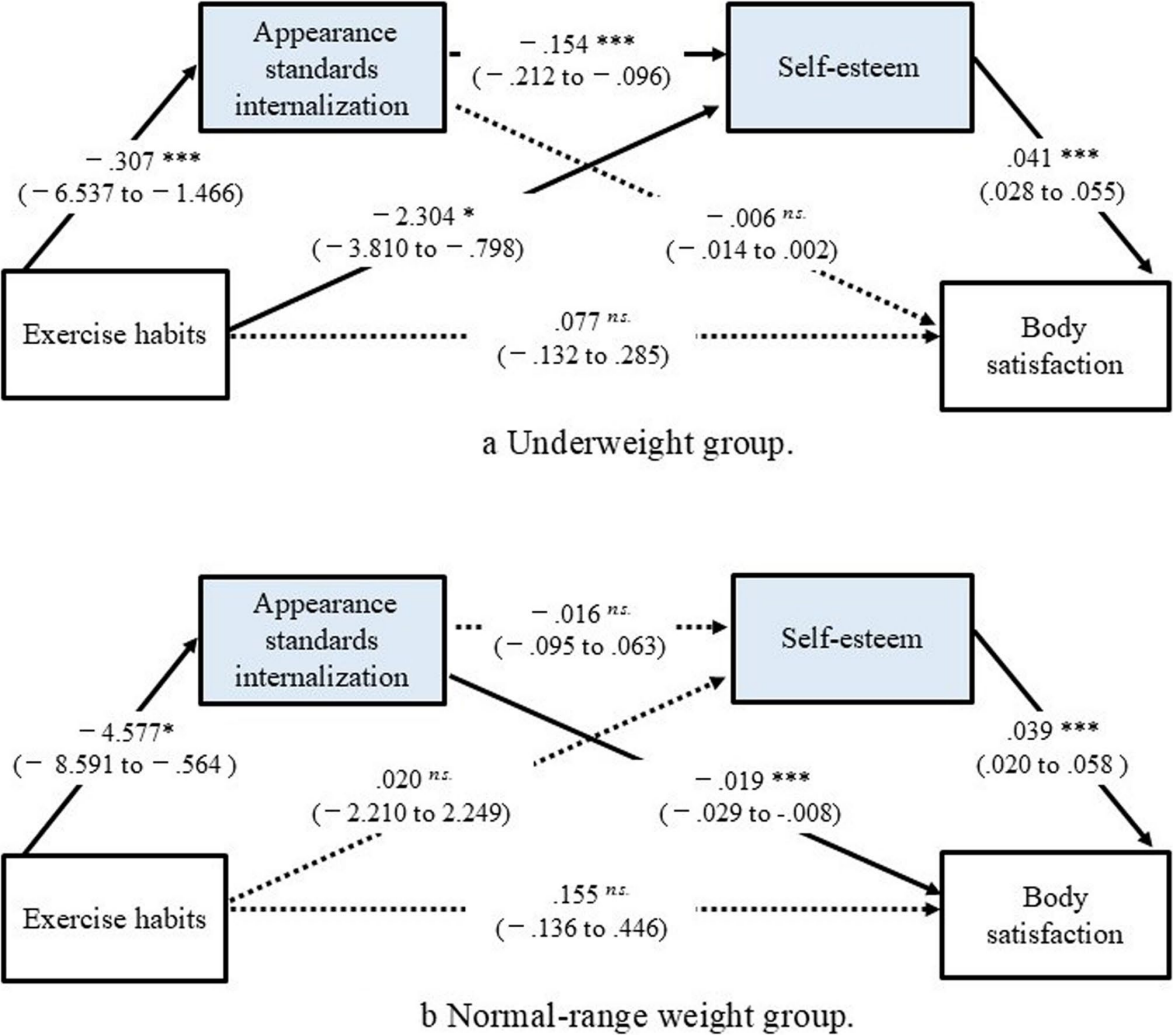
* Note*. The path values presented are the standardized regression coefficients. * *p* < .05, ** *p* < .01, *** *p* < .001. *n.s.*: not significant. Data are standardized coefficients with 95% confidence intervals in parentheses. A solid line indicates a significant effect and a dashed line indicates a non-significant effect

The underweight group demonstrated no direct effect from exercise habits to body satisfaction (B = 0.077, SE = 0.106, 95% CI [−0.132 to 0.285], *p* =.470) (Fig. 2a) Although the total indirect effect was not significant (B = −0.042, BootSE = 0.042, 95% CI [−0.125 to 0.042]), the serial pathway through thin-ideal internalization and self-esteem showed a significant positive indirect effect (B = 0.023, BootSE = 0.010, 95% CI [0.007 to 0.046]). In addition, the pathway through self-esteem alone demonstrated a significant moderate negative indirect effect (B = − 0.088, BootSE = 0.034, 95% CI [−0.161 to − 0.028]).

Focusing on the direct paths, exercise habits had a large and significantly negative effect on thin-ideal internalization (B = −4.002, SE = 1.290, 95% CI [−6.537 to −1.466], *p* <.001) , and a moderate significant negative effect on self-esteem (B = −2.304, SE = 0.766, 95% CI [−3.810 to −0.798], *p* = .003). Thin-ideal internalization also had a large significant negative effect on self-esteem (B = －0.154, SE = 0.029, 95% CI [−0.212 to − 0.096], *p* <.001), while self-esteem had a large significant positive effect on body satisfaction (B = 0.041, SE = 0.007, 95% CI [0.028 to 0.550], *p* <.001).

#### Normal-range weight group

In the normal-range weight group, there was no significant direct effect from exercise habits to body satisfaction (B = 0.155, SE = 0.147, 95% CI [−0.136 to 0.446], *p* =.294) (Fig. 2b), and the total indirect effect was not significant (B = −0.082, BootSE = 0.066, 95% CI [−0.205 to 0.040]). Focusing on each segment of the indirect effect paths, the path from exercise habits to body satisfaction through thin-ideal internalization showed a significant negative indirect effect (B = −0.080, BootSE = 0.045, 95% CI [−0.180 to −0.008]). In contrast, the path through self-esteem alone (B =0.001, BootSE = 0.042, 95% CI [−0.078 to 0.091]) and the serial path through thin-ideal internalization and self-esteem (B = −0.003, BootSE = 0.007, 95% CI [−0.019 to 0.012]) were not significant. For the direct path, exercise habits had a significant positive effect on thin-ideal internalization (B = 4.577, SE = 2.035, 95% CI [8.591 to 0.564], *p* =.026) , but no significant effect on self-esteem (B = 0.020, SE = 1.130, 95% CI [−2.210, 2.249], *p* = .986). Thin-ideal internalization did not significantly predict self-esteem (B = −0.016, SE = 0.040, 95% CI [−0.095, 0.063], *p* = .684). However, thin-ideal internalization had a significantly negative effect on body satisfaction (B = −0.019, SE = 0.005, 95% CI [−0.029 to − 0.008], *p* <.001), while self-esteem had a significant positive effect (B = 0.039, SE = 0.010, 95% CI [0.020 to 0.058], *p* < .001).

## Discussion

This study examined how exercise habits influence body satisfaction through thin-ideal internalization and self-esteem among young, underweight, and normal-range weight women. It focused particularly on underweight women, comparing them with normal-range weight women to uncover potential differences in mechanisms. This research provides unique insights into how thin-ideal internalization and self-esteem affect these groups, contributing valuable evidence for developing strategies to improve young women’s mental and physical health. The following sections provide a discussion of each analysis.

### Comparison of backgrounds characteristics between underweight and normal-range weight groups

Regarding exercise habits, no significant difference was observed between underweight and normal-range weight groups. About 50% of individuals in both groups exercised on average 2.5 days per week. However, the underweight group reported longer exercise durations (1 h and 20 min) than the normal-range weight group (1 h and 6 min), suggesting longer exercise sessions among the underweight group. Both groups had similar reasons for exercising, with over 80% citing “health and fitness” and “perceived lack of exercise” and around 60% citing “beauty enhancement and obesity reduction.”

Research comparing exercise habits by BMI, especially among underweight women, is limited. A survey of university students aged 18 to 35 found that 68% of women exercised at least twice a week, with 30% reporting exercise durations exceeding one hour [12]. Studies have shown that women with a higher BMI engage in exercise more frequently for weight loss and health promotion [13]. This study found that underweight women engage in longer exercise sessions, suggesting differences in motivation and duration between BMI groups. These findings highlight the need for further research on women’s exercise habits and motivations, particularly with a focus on underweight women.

No significant differences were observed between the underweight and normal-range weight groups regarding thin-ideal internalization and self-esteem. However, the underweight group had significantly higher body satisfaction scores, indicating that they perceived themselves as closer to the ideal body shape [20]. Underweight women also tend to engage in longer exercise sessions, which might contribute to their higher body satisfaction. Although studies have shown that women with higher BMIs are more motivated to exercise for weight loss [13], this study found no difference between groups in the percentage of exercising for “beauty enhancement and obesity reduction.” These results highlight the need for further research on women’s exercise habits and motivations with different BMIs, particularly focusing on how underweight women are motivated to engage in active exercise.

### Correlation among measurement variables

A significant negative correlation was observed in the normal-range weight group. In the underweight group, the correlation was also significant but weak. The results align with a meta-analysis showing the strong influence of thin-ideal internalization on body image, especially in normal-range weight women [49]. These findings suggest that a higher level of thin-ideal internalization is associated with lower body satisfaction among women with normal-range weight. A negative correlation was found between thin-ideal internalization and self-esteem in the underweight group but not in the normal-range weight group, suggesting that this relationship differs by BMI.

Studies on young women with normal-range weight and obesity indicate that thin-ideal internalization is unrelated to BMI [50, 51]. However, overweight women with strong thin-ideal internalization show greater concern about their body shape, suggesting a link to BMI [52]. Media-promoted beauty ideals can lead to body dissatisfaction and lower self-esteem [8]. In this context, our study suggests that normal-range weight women with higher thin-ideal internalization and lower self-esteem may benefit from interventions aimed at reducing both groups showed a positive correlation between self-esteem and body satisfaction, consistent with prior evidence that higher body satisfaction strongly correlates with greater self-esteem in women [53]. Furthermore, women with exercise habits tend to have higher self-esteem and body satisfaction [54]. Promoting exercise habits and improving body satisfaction and self-esteem are crucial to promoting young women’s psychological health and well-being. Building on these findings, we next discuss the direct effects of exercise habits on body satisfaction, as identified in the mediation analysis, to evaluate whether these habits independently influence body satisfaction across underweight and normal-range weight groups.

### Direct effects of exercise habits on body satisfaction

The direct effect of exercise habits on body satisfaction was not statistically significant in either group. These results suggest that exercise habits may influence body satisfaction indirectly through other factors. For instance, while exercise frequency is generally associated with positive body image, this relationship can weaken when exercise is primarily motivated by appearance [11]. Furthermore, appearance-driven exercise may diminish the positive effects on body satisfaction, highlighting the complex role of motivation in shaping body image outcomes [11]. Meta-analysis have shown that structured exercise interventions enhance body satisfaction and improve body image [24]. Women with a positive body image often exercise for health and well-being rather than weight loss [55]. They consider exercise as beneficial for health, stress relief, and enjoyment. Participants in this study reported similar exercise motivations, aligning with these findings. However, over 60% of underweight and normal-range weight women cited “beauty enhancement and obesity reduction” as their primary motivation, which may have influenced the impact of exercise on their body satisfaction.

Research suggests that women should avoid exercise emphasizing appearance or weight loss to maintain a positive body image [11]. Another study showed that those committed to exercise have better body image flexibility than those fixated on [54]. Autonomous motivation for exercise is linked to a healthier body image and eating attitudes, thus enhancing influencing body satisfaction [56]. While maintaining appropriate exercise habits is important for young women, other factors are crucial for enhancing body satisfaction. These include reducing thin-ideal internalization and enhancing self-esteem and self-efficacy [11, 26, 57]. Therefore, young women should focus on intrinsic health benefits and stress relief to enhance body satisfaction.

### Indirect effects in the underweight group

In the underweight group, two significant mediation pathways were identified. One showed full mediation, where exercise habits reduced thin-ideal internalization, thereby improving self-esteem and thus enhancing body satisfaction. Research indicates that higher thin-ideal internalization correlates with lower body satisfaction and self-esteem [58]. Our findings support this, indicating that exercise habits are significantly associated with reduced thin-ideal internalization, which, through its indirect effects, mediates improvements in self-esteem and body satisfaction. Another pathway revealed that exercise habits negatively impacted self-esteem, and consequently decreased body satisfaction. While exercise is generally believed to enhance self-esteem [59, 60], evidence suggests it can also negatively affect self-esteem [61]. Some studies have indicated that low BMI strengthens the positive impact of exercise on self-esteem [62, 63]. However, this study did not support this, potentially driven by other variables such as psychological stress or social support. Therefore, the relationship between exercise habits and self-esteem is complex and context-dependent.

Furthermore, unlike the normal-range weight group, the underweight group’s pathway from thin-ideal internalization to body satisfaction was not significant. These results suggest that reducing thin-ideal internalization may not enhance body satisfaction in underweight women. A previous study found that lower thin-ideal internalization is associated with higher body satisfaction and self-esteem [58]. However, different mechanisms may underlie body satisfaction in underweight women. For underweight young women, enhancing body satisfaction may involve addressing thin-ideal internalization and fostering self-esteem through targeted interventions. To further support body satisfaction, psychological interventions aimed at reducing thin-ideal internalization [15] and enhancing self-esteem could play a crucial role [64]. Moreover, for underweight women, developing exercise habits to increase muscle mass is essential for long-term health [65]. These efforts, combined with personalized approaches, can help dispel the misconception that ‘thin is healthy’ and promote a healthier, more fulfilling life as women age.

### Indirect effects in the normal-range weight group

In the normal-range weight group, exercise habits were statistically associated with reduced thin-ideal internalization, which mediated improvements in body satisfaction without directly affecting self-esteem. Higher self-esteem was associated with greater body satisfaction. By contrast, exercise did not mediate this relationship, suggesting that exercise enhances body satisfaction by reducing thin-ideal internalization and improving self-evaluation and self-perception, which is crucial for women with normal-range weight. Research suggests that women’s exercise habits benefit cognitive and mental health, positively influencing self-esteem, body image, and body satisfaction [24, 54–56]. Nonetheless, one study reports that regular exercise has a marginal effect on reducing thin-ideal internalization [66], possibly owing to the importance of appearance in women’s self-concept [67]. Concerning the impact of appearance impact on self-esteem [50, 68], deeper thin-ideal internalization is associated with lower body satisfaction and self-esteem [58].

This study supports the mechanism by which exercise reduces thin-ideal internalization, improving body satisfaction. However, it is noteworthy that the effect of exercise on enhancing self-esteem, as observed in underweight women, was not present. This was likely owing to their lower body satisfaction compared with the underweight group. A higher BMI is associated with significantly lower body satisfaction [20]. Thin-ideal internalization is reinforced by family, peers, and media [6], with women influenced by others’ opinions and media messages from a young age, negatively impacting self-esteem [69]. Especially for young women, social and cultural backgrounds significantly shape self-image [34]; this distorted body image and low body satisfaction triggers unhealthy eating behaviors and physical inactivity [70, 71]. Moreover, thin-ideal internalization is associated with eating and weight concerns [52]. To enhance self-esteem through exercise habits, young women need support from family and friends as well as improvements in self-efficacy [6, 11, 26, 57].

Interventions focusing on factors beyond appearance may be effective for women with normal-range weight. For example, interactive educational programs designed to improve self-esteem have been shown to enhance body image and eating attitudes [72]. It is imperative to introduce universal interventions that foster a critical understanding of beauty standards through media literacy programs and educational systems promoting body positivity and self-esteem [73]. These programs aim to reduce the media’s negative impact and emphasize the body’s functionality over appearance, challenging societal beauty norms and promoting inclusivity.

### Limitations and future directions

This study has some limitations. First, detailed data on the content and intensity of participants’ exercise were not obtained, which limits the understanding of how specific activities influence body satisfaction and self-esteem. Future research should include detailed investigations of exercise content, intensity, frequency, and purpose to elucidate how exercise affects psychological and physical health. Second, the participants’ weight and height were self-reported, which may introduce bias due to unintentional overestimation or underestimation of these values. Such inaccuracies could affect the interpretation of findings, particularly as BMI was used to categorize the sample into groups. Objective measurements are recommended for future studies. Third, the participants were limited to Japanese women, potentially reflecting cultural factors that may not generalize to other populations. Future research should include diverse cultural contexts. Fourth, the study’s sample was not equally balanced between underweight and normal-range weight participants, and future exploration of the motivations behind exercise habits in these groups is needed. Fifth, this study used a single-item measure for assessing body satisfaction. Although this measure was designed for simplicity and to comprehensively capture body satisfaction, its reliability would benefit from comparison with established multi-item scales. Further validation across diverse populations and contexts is warranted. Finally, while mediation analysis identifies potential pathways, it does not fully establish causality. Future research should address confounding factors and ensure temporal precedence to draw causal inferences.

## Conclusion

This study’s main finding is that underweight young women’s exercise habits affect body satisfaction mediated by thin-ideal internalization and self-esteem, which differs from the pattern observed in women with normal-weight. For underweight women, exercise habits reduce thin-ideal internalization, enhancing self-esteem and improving body satisfaction. Maintaining regular exercise habits is essential for improving body satisfaction and overall health. The findings underscore the importance of promoting exercise in a balanced and healthy lifestyle. Research has not adequately addressed approaches for underweight women, and this study fills this gap. Practical applications include body image education, health education, and psychological support. Programs should aim to reduce thin-ideal internalization and promote exercise habits to enhance self-esteem, while also provide concrete guidelines to mitigate media influence while supporting a healthy body image and improved body satisfaction.

In contrast, while exercise habits reduced thin-ideal internalization and improved body satisfaction for women with normal-range weight, they did not influence self-esteem. The results suggest that maintaining exercise habits is crucial, but additional approaches are needed to enhance self-esteem in women with normal-range weight. A multifaceted approach is proposed, including body image education, media literacy, and self-awareness improvement. These could lead to programs focusing on factors beyond appearance, contributing to improved body satisfaction and self-esteem among these women. Future research should include longitudinal and interventional studies to clarify the causal relationships between exercise habits, body satisfaction, and self-esteem. Additionally, examining similar mechanisms across other BMI categories, including obesity, is essential. This study provides a foundation for tailored health education for women with different BMIs, highlighting the need for new approaches, particularly for underweight women. This initiative will ultimately improve young women’s mental and physical health, enabling each individual to choose and maintain a healthy, comfortable, and authentic body—a truly “well body.”

## Data Availability

The datasets used and/or analyzed during the current study are available from the corresponding author on reasonable request.

## Abbreviations

BMI: Body mass index
Underweight: BMI≤18.5kg/m²
Normal-range weight: BMI 18.5–25 kg/m²
Obese: BMI≥25kg/m²
SATAQ-3 JS: Sociocultural Attitudes Towards Appearance Questionnaire-3 Japanese version
RSE-J: Rosenberg Self-Esteem Scale Japanese versionRosenberg Self-Esteem Scale Japanese version
